# Similarities and differences between multiple sclerosis and type 1 diabetes

**DOI:** 10.1002/dmrr.3505

**Published:** 2021-10-22

**Authors:** Valeria Pozzilli, Eleonora Agata Grasso, Valentina Tomassini

**Affiliations:** ^1^ Department of Neurosciences, Imaging and Clinical Sciences Institute of Advanced Biomedical Technologies (ITAB) University “G. d’Annunzio” of Chieti‐Pescara Chieti Italy; ^2^ Department of Clinical Neurology Multiple Sclerosis Centre SS. Annunziata University Hospital Chieti Italy; ^3^ Department of Paediatrics SS. Annunziata University Hospital Chieti Italy

**Keywords:** autoimmunity, comorbidity, COVID‐19, multiple sclerosis, SARS‐CoV‐2 infection, type 1 diabetes

## Abstract

Multiple sclerosis (MS) and type 1 diabetes (T1D) are chronic conditions that result from dysfunction of the immune system. Their common root in autoimmunity stimulates interest in the exploration of similarities and differences between the two diseases. Genetic susceptibility is relevant, creating a substrate, on which environmental factors act as a trigger of an aberrant immune response. Despite being both T‐cell mediated disorders with a strong involvement of the humoral arm, immunomodulation is a mainstay of MS management, whereas hormone replacement therapy remains the principal approach for T1D. T1D is usually diagnosed in children and adolescents, while MS is typical of young adults. This difference has implications for disease progression and treatment. The SARS‐CoV‐2 pandemic and its effect on immunity may affect the prevalence of these conditions, as well as their clinical manifestation.

## 1 INTRODUCTION

**FIGURE 1:**
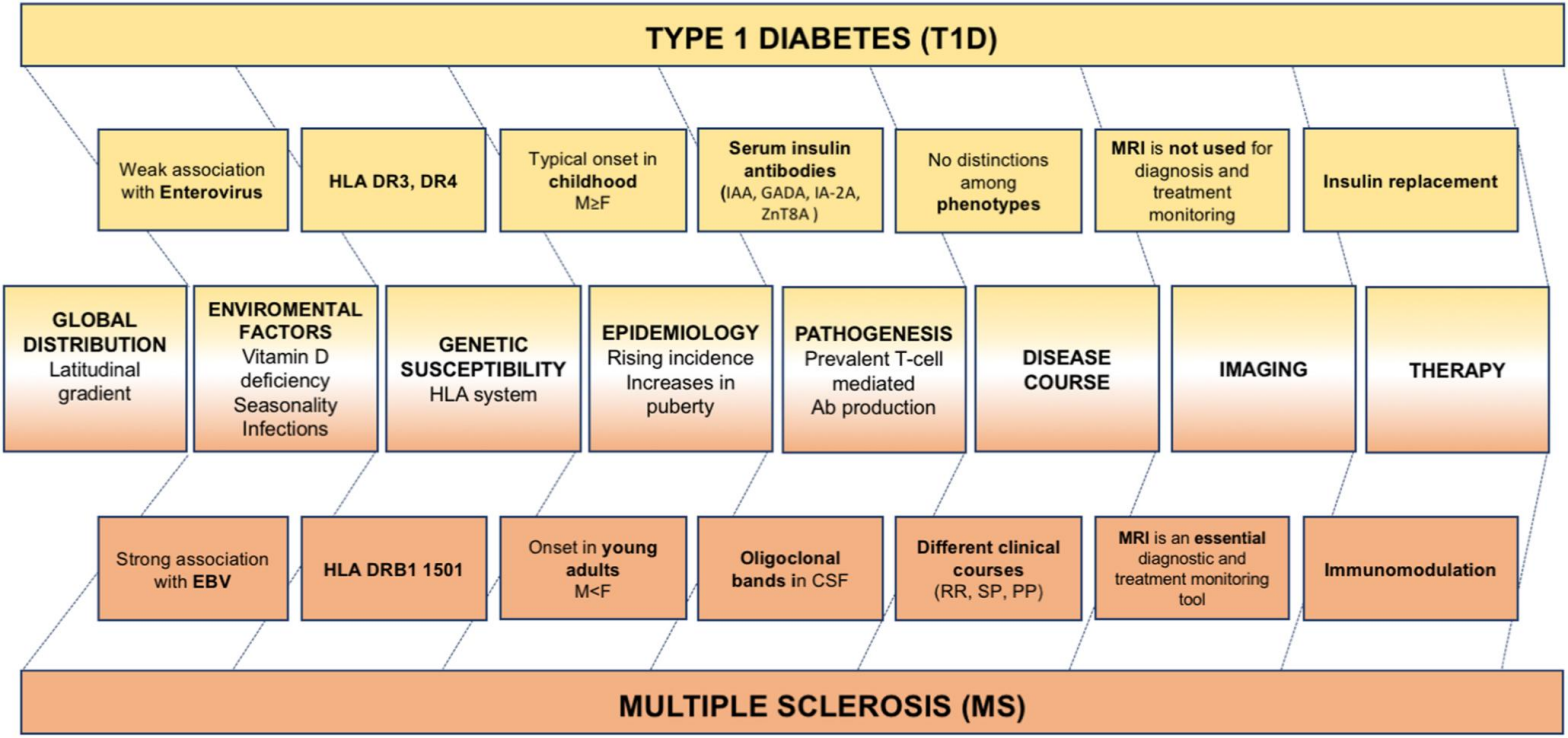
Similarities and differences between type 1 diabetes and multiple sclerosis. The middle row shows commonalities between the two conditions; differences or characteristics specific to one condition only are reported in the rows close to the each of the conditions. CSF, cerebrospinal fluid; CT, computed tomography; EBV, Epstein–Barr virus; GADA, glutamic acid decarboxylase antibody; HLA, human leucocyte antigen; IA‐2A, islet antigen 2 autoantibody; IAA, insulin autoantibodies; MRI, magnetic resonance imaging; PP, primary progressive; RR, relapsing remitting; SP, secondary progressive; ZnT8A, zinc transporter 8 autoantibody

Both multiple sclerosis (MS) and type 1 diabetes (T1D) are chronic conditions that result from dysfunction of the immune system. They may co‐occur: a threefold higher incidence of MS has been observed in patients affected by T1D in comparison to the general population.^1^ Despite differences in demographics at disease onset and in clinical characteristics, their common root in autoimmunity has led researchers to discover commonalities in genetic, environmental and immunological features (Figure 1).

## 2 GENETIC SUSCEPTIBILITY AND ENVIRONMENTAL FACTORS

Genetic susceptibility is relevant in both MS and T1D.^2^ Sharing of haplotypes is unlikely, considering that the human leucocyte antigen haplotype DRB1*1501‐DQA1*0102‐B1*0602 confers susceptibility for MS, but protects against T1D.^3^ However, a population‐based study in Sardinia described a fivefold greater prevalence of T1D in MS patients when compared to the general population, explaining this phenomenon through the presence, in this unique population, of DRB1*0405‐DQA1*0501‐DQB1*0301 and DRB1*0301‐DQA1*0501‐DQB1*0201 haplotypes, which were found to increase the risk for both conditions.^4^ Additionally, single nucleotide polymorphisms (SNPs) are believed to contribute to the genetic susceptibility for both diseases and an overlap between SNPs has been described. Indeed, evidence from loci of susceptibility suggest that two out of seven SNPs (rs12708716 from the *CLEC16A* gene and rs763361 from the *CD226* gene) known to be associated with T1D are also associated with MS (Table 1). These findings prove, on one hand, the polygenic modality of inheritance for both conditions and, on the other, suggest a common root for MS and T1D.^5^

**TABLE 1:** Similarities and differences between MS and T1D in age, genetic loci, autoimmunity and treatment targets

	MS	T1D
Typical age at onset	20–40 years old (12)	<20 years old (23)
Predisposing HLA worldwide	DRB1*1501‐DQA1*0102‐B1*0602 (3)	DRB1*03:01‐DQA1*05:01‐DQB1*02:01 (abbreviated “DR3”) and DRB1*04:01/02/04/05/08‐DQA1*03:01‐DQB1*03:02/04 (or DQB1*02; abbreviated “DR4”) (38)
Predisposing HLA in the Sardinian population	DRB1*0405‐DQA1*0501‐DQB1*0301 and DRB1*0301‐DQA1*0501‐DQB1*0201 (4)	
Common SNPs	rs12708716 from the *CLEC16A* gene, rs763361 from the *CD226* gene (5)	
Autoimmunity involvement	T cells and oligoclonal bands (13)	T cells and autoantibodies (11)
Immunological targets for treatments	CD20, α4β1‐integrin, CD52, S1P1‐R, DNA (24)	CD20, CD3, CTLA‐4, LFA3, IL‐1 (15)

Abbreviations: HLA, human leucocyte antigen; SNPs, single nucleotide polymorphisms.

The genotype variability only partially explains the co‐occurrence of MS and T1D, suggesting that other factors are also at play.^6^ In fact, MS and T1D share some environmental factors that are thought to be involved in their pathogeneses. A latitudinal gradient is present for both conditions. Their increased prevalence away from the equator^7^ suggests that vitamin D may be involved.^8^, ^9^ Viruses, such as Enterovirus (e.g., Coxsackievirus) in T1D and EBV in MS, are considered a possible trigger, leading to molecular mimicry in both conditions.^10^

## 3 IMMUNE PATHOPHYSIOLOGY AND TREATMENT

Regardless of the specific predisposing or triggering factors inducing the immune response, both MS and T1D are T‐cell mediated disorders with a strong involvement of the B‐cell compartment. T1D results from targeted destruction of pancreatic β cells by T cells, activated after recognition of specific insulin epitopes on antigen presenting cells. Almost all children with two or more insulin antibodies (IAA, GADA, IA‐2A, ZnT8A) will develop clinical T1D over time.^11^ MS develops within the central nervous system, as a result of an aberrant peripheral T‐cell activation after antigen presentation. Myelin‐related antigens, such as myelin basic protein and myelin oligondendrocyte glycoprotein, are thought to play a role in the pathogenesis of the disease.^12^ However, oligoclonal bands are the immunological hallmarks that can be found with high sensitivity in more than 85% of MS patients (Table 1).^13^ Both in MS and in T1D the mechanism of epitope spreading, with a shifting of T‐cell autoreactivity from primary initiating self‐determinants to defined cascades of secondary determinants, has been claimed to explain sustained inflammatory processes during disease progression.^14^

Despite its relatively well‐defined pathogenesis, the main treatment for T1D is hormone replacement, whereas MS therapy is based on immunomodulation or selective immunosuppression. As for many other autoimmune conditions, disease‐modifying therapies (DMTs) are the staple of MS treatment, with several medications used to modulate the immune system by targeting its adaptive and humoral branches (Table 1). A similar approach is being evaluated for the treatment of T1D.^15^ For example, the CTLA4‐Ig Abatacept, used in rheumatological conditions,^16^ can preserve C‐peptide levels and improve insulin sensitivity for a limited interval of time (i.e., 48 months).^17^ Evidence from targeting B cells with an anti CD20 monoclonal antibody showed that a course of treatment can delay the fall of C‐peptide by months.^18^ Whereas anti CD 20 monoclonal antibodies are not commonly used in T1D, they are used in MS to achieve a reduction of disease activity and a delay of disease progression.^19^

## 4 AGE OF ONSET AND ITS IMPLICATIONS

The age of onset of T1D is normally earlier than MS and, in both conditions, can affect sex differences in the prevalence, disease course and prognosis.^20^, ^21^ T1D is usually diagnosed in patients above the age of 4, although some cases of T1D can be discovered in adulthood and misdiagnosed as type 2 diabetes.^22^ On the other hand, paediatric onset of MS (POMS) represents only 3%–10% of all MS diagnoses, with only 10%–20% of POMS diagnosed before the age of 10^23^; as for T1D, the diagnosis of MS can occur later in life, despite a paediatric onset (Table 1). The incidence of both conditions increases at puberty.^22^, ^23^ In MS, it continues to increase in young adults,^23^ whereas the incidence of T1D stabilises.^22^ Unlike the female preponderance typical of adult onset MS,^23^ in T1D, there is a slightly higher prevalence of males.^22^

In POMS, the co‐occurrence of the two diseases may have therapeutic implications for a personalised approach to therapy. Indeed, POMS tends be actively inflamed, when compared to adult onset MS, and thus requires more aggressive immunotherapy^24^ that may influence the immunological mechanisms contributing to T1D. Currently, there are only a few immunomodulatory treatments approved for paediatric‐onset MS^25^ and thus the therapeutic implications of T1D and MS comorbidity remain to be explored.

Age has important implications also for the clinical evolution and therapeutic management of both conditions. An earlier age of MS onset is typically associated with a relapsing course and longer time to conversion to secondary progression.^26^ In T1D, instead, an earlier diagnosis is associated with a higher risk of clinical progression^27^: β cells are not capable of regeneration after destruction and this may, at least in part, explain differences in the prognosis of the condition. The later disease onset in MS than in T1D may be facilitated by brain plasticity that limits the clinical manifestation of MS damage. Indeed, T1D diagnosis usually occurs after a consistent loss of β cells, while, in MS, the time of diagnosis does not correspond to an exhaustion of compensatory mechanisms.^28^

The immune response is an age dependent process that can affect response to treatment in both T1D and MS. A younger age at MS onset is associated with better response to immunomodulation because of a higher inflammatory activity.^23^ Similarly, in newly diagnosed T1D, a short course with the anti‐CD3 Teplizumab, leads to a preservation in C‐peptide levels in the medium term and this effect is greater in younger patients.^29^ Recent studies have shown that Teplizumab can improve and stabilise β cell function in antibody‐positive high‐risk individuals, therefore delaying the onset of T1D.^30^

## 5 MS AND T1D IN THE ERA OF THE SEVERE ACUTE RESPIRATORY SYNDROME CORONAVIRUS 2 (SARS‐CoV‐2) PANDEMIC

The SARS‐CoV‐2 infection that caused the coronavirus disease 2019 (COVID‐19) pandemic may have consequences both in MS and in T1D. Diabetes has been considered as a risk factor for a rapid progression and worse prognosis of COVID‐19,^31^ possibly because of an inflammatory state induced by the virus. Similarly, it has been debated whether MS is a risk factor for SARS‐CoV‐2 infection or for its evolution, especially in the light of the widespread use of DMTs in these patients, despite current evidence suggests that DMTs for MS have an acceptable level of safety.^32^ Risk factors associated with worse clinical severity of SARS‐CoV‐2 infection in MS patients seem to be high levels of disability, older age, black race and recent treatment with corticosteroids.^33^ Also, a reduced access to medical care during the pandemic may have contributed to late diagnosis and worse presentation of both conditions.^34^, ^35^

One can speculate that SARS‐COV‐2 infection may trigger an autoimmune response towards neurons by exposing new neural or vascular antigens, as it may occur for SARS‐CoV‐2 infection of the pancreas, which might trigger a β‐cell autoimmunity in the long‐term.^36^ Considering the pandemic of COVID‐19, an increased prevalence of autoimmune conditions, possibly with new phenotypic features, may appear in the future.

## 6 CONCLUSIONS

T1D and MS share some commonalities and present some differences, specific to the systems that they target. Exploiting research evidence from one condition, in which identification of biomarkers for early diagnosis and development of targeted treatment approaches have improved quality of life, may stimulate similar improvements for the other condition. A deeper understanding of the commonalities and differences between autoimmune conditions will accelerate progress, by cross‐fertilisation of knowledge, towards early diagnosis and effective personalised management. The current pandemic poses new challenges for the management of these conditions and may lead to an increase in their prevalence, possibly with novel phenotypes, in the future.

## CONFLICTS OF INTEREST

The authors have no conflict of interest to report.

## ETHICAL APPROVAL

For this article, Ethical Committee approval was not required.

## AUTHOR CONTRIBUTION

Valeria Pozzilli, Eleonora Agata Grasso and Valentina Tomassini all contributed to manuscript preparation.

### PEER REVIEW

The peer review history for this article is available at https://publons.com/publon/10.1002/dmrr.3505.

## Data Availability

Data sharing is not applicable to this article, as no datasets were generated or analysed for this work.
